# System Economic Costs of Antibiotic Use Elimination in the US Beef Supply Chain

**DOI:** 10.3389/fvets.2021.606810

**Published:** 2021-04-26

**Authors:** Karun Kaniyamattam, Loren W. Tauer, Yrjö T. Gröhn

**Affiliations:** ^1^Department of Population Medicine and Diagnostic Sciences, Cornell College of Veterinary Medicine, Ithaca, NY, United States; ^2^Charles H. Dyson School of Applied Economics and Management, Cornell University College of Agriculture and Life Sciences and Cornell SC Johnson College of Business, Ithaca, NY, United States

**Keywords:** economic modeling, linear programming, antibiotic use, antimicrobial resistance, US beef production system

## Abstract

There is consumer pressure on the US beef cattle industry to minimize antibiotic use (ABU) in order to aid in the global antimicrobial resistance mitigation efforts. Our objective was to estimate the economic costs of ABU constraints in a conceptual US integrated beef supply chain (IBSC) to aid the beef industry in mitigating the ever-increasing risk of antimicrobial resistance, by reducing their ABU. An IBSC network model was developed and differentiated into 37 different nodes of production. Each node could only raise a specific type of animals, differentiated based on the production technique and animal health status. The cost, as well as the weight gain coefficient, was estimated for each node, using an IBSC cost of production model. Linear programming solutions to this network model provided the least cost path of beef supply through the system, under various ABU constraints. The cost as well as weight gain coefficient of the 37 nodes, initial supply of 28.5 million calves weighing 0.65 million metric tons, and final demand of 16.14 million metric tons of slaughter-ready fed cattle were used as inputs/constraints to the three different linear programming scenarios, with different ABU constraints. Our first scenario, which placed no constraint on ABU, estimated that the minimum total economic cost to meet the final beef demand was $38.6 billion. The optimal solution was to use only the high health status calves for beef production. Because low health calves occur in the beef system, our second scenario required all the calves irrespective of their health status to be used, which increased the system cost to $41.5 billion. Thus, the value of only producing high health status calves is $2.9 billion. Our third scenario, which restricted feedlots from using antibiotics even for low health calves, incurred a total cost of $41.9 billion for antibiotic-free beef production. We concluded that the additional cost of $367 million for implementing antibiotic-free beef production is relatively low, ~0.90% of the minimum cost incurred for the conventional beef supply chain (model 2 cost of $41.5 billion). However, a much higher cost savings is obtained by producing only high health status calves.

## Introduction

The $68 billion (1) US beef supply chain is one of the leading consumers of medically important antibiotics, with an annual average purchase of ~2,500 tons (2). Antibiotics are used by the beef industry to prevent and treat the major beef cattle diseases (3) such as bovine respiratory diseases (BRDs), liver abscess, and lameness. The recent USDA survey on antimicrobial use (AMU) in US feedlots reported that about 56% of feedlots (4) used medically important antimicrobials. The most commonly used antibiotics in beef systems were tetracyclines, followed by macrolides (2). Phenicols, beta-lactams, fluoroquinolones, and sulfonamides were also used for treatment and metaphylaxis of BRD. In comparison to feedlots, AMU in cow–calf operations was relatively low at <20% of herds. Human interactions with the beef systems either through unhygienic meat consumption (5, 6) or contact with environmental effluents (7) are shown to be a significant risk for the transmission and persistence of antimicrobial resistance (AMR) in humans. The ever-increasing risk of AMR is placing pressure on beef systems to reduce their antibiotic use (ABU), even at the expense of lower weight gain efficiency. However, there are only a limited number of studies that investigate the economic effects of antibiotic elimination from beef production. Recently Dennis et al. (8) estimated the value of metaphylaxis (preventive antimicrobial treatment used in feedlots) to be at least $532 million annually for the entire US feedlot industry. However, they limited their economic analysis to the feedlots sector of the US beef system.

A significant characteristic of the US beef system is the vast structural and regional differences in the size of operations, cost of production, and health management practices as detailed in Lhermie et al. (9). The 26 million beef cattle head slaughtered annually is sourced through 913,246 cattle and calf operations (10) spread across the United States. Essentially, the US beef system, which produced 12.2 million metric ton (MMT) of beef (10) in year 2019, can be envisaged as a distributed supply chain from farm to table. Approximately 28 million beef calves are born every year in the United States (9), which are weaned at an average age of 5 months. Depending on the season of weaning, there are at least five different intermediate sector management practices in beef production through which these calves are routed. Feeder cattle from these intermediate production sectors are sent to feedlots, where they are kept until they reach slaughter weight. Frequent changes in ownership of beef cattle ranging from farmers through feedlot operators may provide disincentives for the adoption of practices that can reduce AMU (9).

The trend over recent years across all food animal production systems is vertical integration or consolidation of production (11) sectors. One advantage to integrating the various sectors of production is better data capture especially with respect to health management practices. Recent trends in beef systems have shown an increase in interest in integrated beef supply chains (IBSCs) (12, 13), which enables traceability throughout the entire chain (14) with respect to the health of animals. Various feedlot health management strategies with respect to ABU are available to the beef industry at present. If the feeder cattle arriving at the feedlot are known to be of low health status, the entire herd is often subjected to metaphylaxis. The other end of the spectrum with respect to ABU strategy is grass-finished free-ranging cattle, which may never be subjected to ABU. Hence, our objective was to investigate the movement of cattle through the US beef system with the use and non-use of various antibiotic health management strategies. This is accomplished by constructing a conceptual network model of an IBSC, which tracks the health status of animals and subsequently the ABU strategy throughout the supply chain. Our conceptual model enables us to calculate the costs, as well as weight gain coefficients of channelizing beef through different production, as well as health management strategies. Linear programming (LP) optimization applied to our network model determines the cattle supply through the beef production system that results in minimum economic costs to the IBSC network model under various ABU constraints.

## Materials and Methods

### Key Production and Health Management Features of the US Beef Supply Chain

#### Cow-Calf Operations

The US beef industry comprised 727,906 cow–calf operations (10). The cow–calf operations can be divided into three groups based on herd size into small (<50), medium (50–199), and large (>200). Percentages of herds that provide calf buyers with information about calf-health program among small, medium, and large here were 35, 60, and 79%, respectively (15).

#### Stocker/Backgrounding Operations

These operations are located across the United States in different geographical locations. Distinct locations have an effect on the seasonal availability of feed, which explains the five different intermediate steps in beef production to which the weaned calves are subjected to, namely, (1) preconditioning, (2) backgrounding, (3) winter grazing, (4) dry lot winter stocking, and (5) some calves with sufficient average daily gain (ADG) are directly shipped to feedlots (16). Preconditioning, a health management practice that keeps animals healthy by optimal weaning, vaccination, deworming, and dehorning, as well as feed transition to the feedlot, is shown to improve health of cattle and has significant impact on the intermediate sector profit (17). The stockers are predominantly grass-fed, whereas backgrounders are fed on a transitioning ration, which aim to acclimatize feeder cattle to subsequent feedlot phase. Large stocker or backgrounding enterprises (>500 head) vs. small enterprises (<100 head) are more likely (18) to use modified live vaccines (88 vs. 44%), as well as preventive approaches to ticks and internal parasites (90 vs. 81%).

#### Feedlot Operations

Feeder cattle from these intermediate production sectors are sent to feedlots, across different seasons within a year. Approximately 81% of the US fed cattle (11.6 million out of 14.4 million) are raised by commercial feedlots with 1,000-head capacity or more (19). Depending on the feedlot placement weight, seasonal availability of feed, and market fluctuations in fed cattle prices, the feeder cattle are kept anywhere between 100 and 230 days in the feedlots until they reach slaughter weight (16). Approximately 95% of commercial feedlots employ respiratory diseases vaccines, 72% employ clostridial vaccines, and 87% treat their cattle for parasites (20). Approximately 88% of feedlots gave antimicrobials in feed, water, or by injection in year 2016 (4) for prevention, control, or treatment of BRD or coccidiosis and for growth promotion. Approximately 56% of the feedlots used some of the medically important antimicrobials in the feed (4). Approximately 2,500 tons (a 30% reduction from 2016 sales) of medically important antimicrobials (2) were marketed to the US cattle industry in 2018, even after US Food and Drug Administration's Guidance for Industry #213 (21), which banned the use of medically important antimicrobials for growth promotion in food animal production.

### The IBSC Network Model

**Figure 1:**
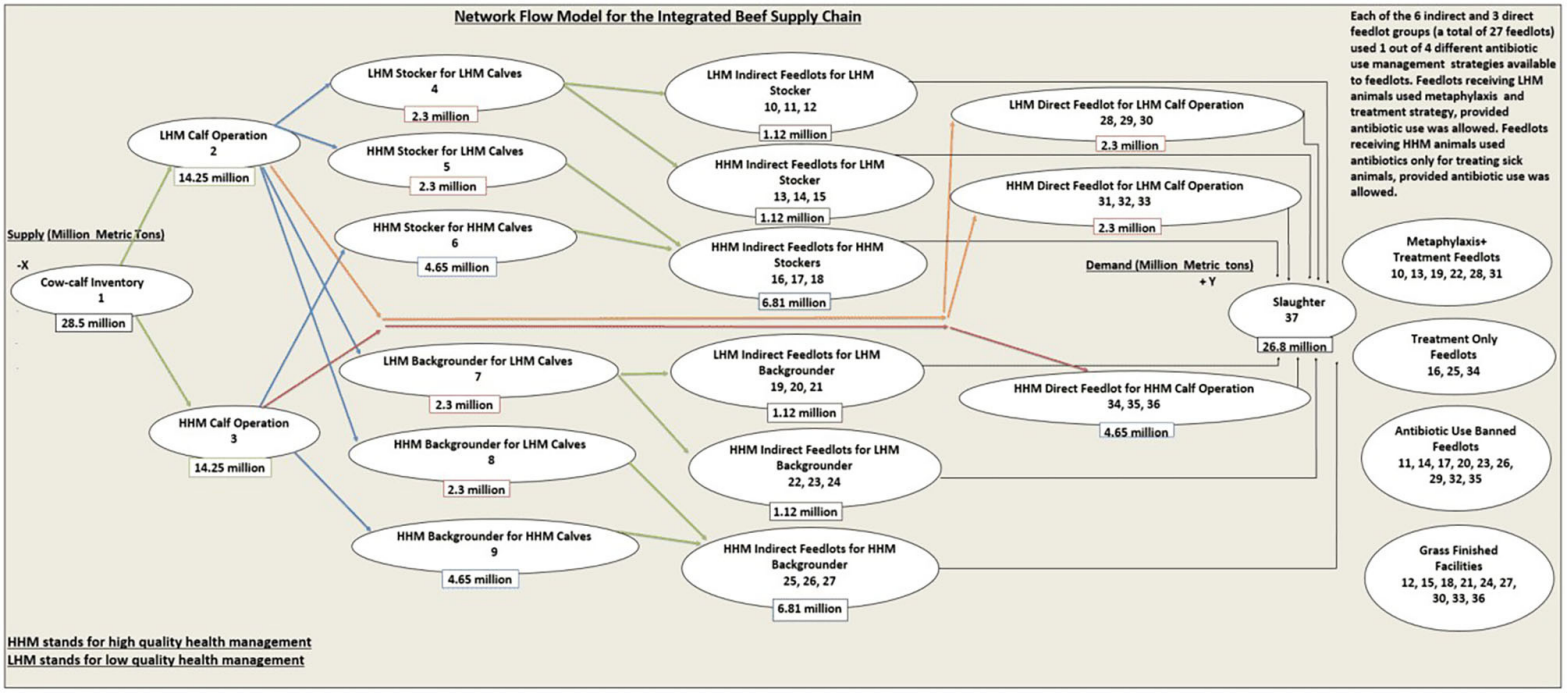
The schematic representation of the conceptual network flow model for the integrated beef supply chain representing the entire United States beef production system.

In order to mimic the production as well as health management practices inherent in the US beef supply chain, we developed a conceptual IBSC (22, 23) network model, which has 37 different nodes of production (Figure 1). The cow–calf inventory (node 1) supplies two types of calves [reared with low-quality health management (LHM) or with high-quality health management (HHM)] to LHM calf operation (node 2) and HHM calf operation (node 3), respectively, classified based on the inherent quality of health. The distinction between LHM and HHM animals was assumed to be subjective, based on the criteria utilized to define health status. This distinction can vary between production systems based on the management practices and genetics of health traits that a decision maker is interested in [e.g., disease resistance to BRD (24) vs. a composite health trait (25) that accounts for the general performance of animal across the beef supply chain]. A total of 68 possible node movements through the system were possible (Table 1) based on the logic we used for this IBSC network model, which is explained below.

**Table 1:** The solutions for the three linear programming scenarios^a^ applied to integrated beef supply chain network model, each with a different antibiotic use demand constraint.

			**Scenario 1. Basic optimization**				**Scenario 2. Calf supply constrained**				**Scenario 3. Antibiotic use constrained**			
			**No constraints on cow–calf, stocker, or feedlot sector**				**Cow–calf inventory has to supply equal amount calves to both calf operations**				**All indirect and direct feedlots using antibiotics are shut down**			
**From–>to (node)**	**Weight gain coefficient (%)**	**Cost^b^ (per metric ton)**	**Beef flow-out^c^**	**Beef flow-in^c^**	**Total cost^b^**	^*^Opportunity cost^b^	**Beef flow-out^c^**	**Beef flow-in^c^**	**Total cost^b^**	^*^Opportunity cost^b^	**Beef flow-out^c^**	**Beef flow-in^c^**	**Total cost^b^**	^*^Opportunity cost^b^
**1–>2**	100	$3,744	**—**	**—**	**—**	**$0**	**0.32**	**0.32**	**$1,211**	**$0**	**0.32**	**0.32**	**$1,211**	**$0**
**1–>3**	100	$3,744	**0.57**	**0.57**	**$2,146**	**$0**	**0.32**	**0.32**	**$1,211**	**$0**	**0.32**	**0.32**	**$1,211**	**$0**
**2–>4**	1,051	$4,472	**-**	**-**	**-**	**$0**	**—**	**—**	**—**	**$0**	**—**	**—**	**—**	**$0**
**2–>5**	1,051	$4,472	**—**	**—**	**—**	**$0**	**—**	**—**	**—**	**$0**	**—**	**—**	**—**	**$0**
**2–>7**	1,051	$4,472	**—**	**—**	**—**	**$0**	**—**	**—**	**—**	**$0**	**—**	**—**	**—**	**$0**
**2–>8**	1,051	$4,472	**—**	**—**	**—**	**$0**	**—**	**—**	**—**	**$0**	**—**	**—**	**—**	**$0**
**2–>28**	1,051	$4,472	**—**	**—**	**—**	**$0**	**0.27**	**2.84**	**$11,472**	**$0**	**—**	**—**	**—**	**$0**
**2–>29**	1,051	$4,472	**—**	**—**	**—**	**$0**	**—**	**—**	**—**	**$0**	**0.27**	**2.84**	**$11,472**	**$0**
**2–>30**	1,051	$4,472	**—**	**—**	**—**	**$0**	**—**	**—**	**—**	**$0**	**—**	**—**	**—**	**$0**
**2–>31**	1,051	$4,472	**—**	**—**	**—**	**$0**	**—**	**—**	**—**	**$0**	**—**	**—**	**—**	**$0**
**2–>32**	1,051	$4,472	**—**	**—**	**—**	**$0**	**—**	**—**	**—**	**$0**	**—**	**—**	**—**	**$0**
**2–>33**	1,051	$4,472	**—**	**—**	**—**	**$0**	**—**	**—**	**—**	**$0**	**—**	**—**	**—**	**$0**
**3–>6**	1,180	$4,151	**0.57**	**6.77**	**$25,703**	**$0**	**0.32**	**3.82**	**$14,507**	**$0**	**0.32**	**3.82**	**$14,507**	**$0**
**3–>9**	1,180	$4,151	**—**	**—**	**—**	**$0**	**—**	**—**	**—**	**$0**	**—**	**—**	**—**	**$0**
**3–>34**	1,180	$4,151	**—**	**—**	**—**	**$0**	**—**	**—**	**—**	**$0**	**—**	**—**	**—**	**$0**
**3–>35**	1,180	$4,151	**—**	**—**	**—**	**$0**	**—**	**—**	**—**	**$0**	**—**	**—**	**—**	**$0**
**3–>36**	1,180	$4,151	**—**	**—**	**—**	**$0**	**—**	**—**	**—**	**$0**	**—**	**—**	**—**	**$0**
**4–>10**	134	$3,737	**—**	**—**	**—**	**$0**	**—**	**—**	**—**	**$0**	**—**	**—**	**—**	**$0**
**4–>11**	134	$3,737	**—**	**—**	**—**	**$0**	**—**	**—**	**—**	**$0**	**—**	**—**	**—**	**$0**
**4–>12**	134	$3,737	**—**	**—**	**—**	**$0**	**—**	**—**	**—**	**$0**	**—**	**—**	**—**	**$0**
**4–>13**	134	$3,737	**—**	**—**	**—**	**$0**	**—**	**—**	**—**	**$0**	**—**	**—**	**—**	**$0**
**4–>14**	134	$3,737	**—**	**—**	**—**	**$0**	**—**	**—**	**—**	**$0**	**—**	**—**	**—**	**$0**
**4–>15**	134	$3,737	**—**	**—**	**—**	**$0**	**—**	**—**	**—**	**$0**	**—**	**—**	**—**	**$0**
**5–>16**	153	$3,023	**—**	**—**	**—**	**$1,454**	**—**	**—**	**—**	**$438**	**—**	**—**	**—**	**$349**
**5–>17**	153	$3,023	**—**	**—**	**—**	**$1,454**	**—**	**—**	**—**	**$438**	**—**	**—**	**—**	**$349**
**5–>18**	153	$3,023	**—**	**—**	**—**	**$1,454**	**—**	**—**	**—**	**$438**	**—**	**—**	**—**	**$349**
**6–>16**	165	$1,206	**6.77**	**11.13**	**$5,263**	**$0**	**3.82**	**6.28**	**$2,971**	**$0**	**—**	**—**	**—**	**$0**
**6–>17**	165	$1,206	**—**	**—**	**—**	**$0**	**—**	**—**	**—**	**$0**	**3.82**	**6.28**	**$2,971**	**$0**
**6–>18**	165	$1,206	**—**	**—**	**—**	**$0**	**—**	**—**	**—**	**$0**	**—**	**—**	**—**	**$0**
**7–>19**	134	$3,991	**—**	**—**	**—**	**$0**	**—**	**—**	**—**	**$0**	**—**	**—**	**—**	**$0**
**7–>20**	134	$3,991	**—**	**—**	**—**	**$0**	**—**	**—**	**—**	**$0**	**—**	**—**	**—**	**$0**
**7–>21**	134	$3,991	**—**	**—**	**—**	**$0**	**—**	**—**	**—**	**$0**	**—**	**—**	**—**	**$0**
**7–>22**	134	$3,991	**—**	**—**	**—**	**$0**	**—**	**—**	**—**	**$0**	**—**	**—**	**—**	**$0**
**7–>23**	134	$3,991	**—**	**—**	**—**	**$0**	**—**	**—**	**—**	**$0**	**—**	**—**	**—**	**$0**
**7–>24**	134	$3,991	**—**	**—**	**—**	**$0**	**—**	**—**	**—**	**$0**	**—**	**—**	**—**	**$0**
**8–>25**	153	$3,185	**—**	**—**	**—**	**$1,505**	**—**	**—**	**—**	**$489**	**—**	**—**	**—**	**$400**
**8–>26**	153	$3,185	**—**	**—**	**—**	**$1,505**	**—**	**—**	**—**	**$489**	**—**	**—**	**—**	**$400**
**8–>27**	153	$3,185	**—**	**—**	**—**	**$1,505**	**—**	**—**	**—**	**$489**	**—**	**—**	**—**	**$400**
**9–>25**	165	$1,264	**—**	**—**	**—**	**$0**	**—**	**—**	**—**	**$0**	**—**	**—**	**—**	**$0**
**9–>26**	165	$1,264	**—**	**—**	**—**	**$0**	**—**	**—**	**—**	**$0**	**—**	**—**	**—**	**$0**
**9–>27**	165	$1,264	**—**	**—**	**—**	**$0**	**—**	**—**	**—**	**$0**	**—**	**—**	**—**	**$0**
**10–>37**	185	$3,458	**—**	**—**	**—**	**$2,794**	**—**	**—**	**—**	**$1,980**	**—**	**—**	**—**	**$1,896**
**11–>37**	185	$4,054	**—**	**—**	**—**	**$3,301**	**—**	**—**	**—**	**$2,486**	**—**	**—**	**—**	**$2,403**
**12–>37**	185	$4,275	**—**	**—**	**—**	**$3,489**	**—**	**—**	**—**	**$2,674**	**—**	**—**	**—**	**$2,590**
**13–>37**	185	$3,458	**—**	**—**	**—**	**$2,794**	**—**	**—**	**—**	**$1,980**	**—**	**—**	**—**	**$1,896**
**14–>37**	185	$4,054	**—**	**—**	**—**	**$3,301**	**—**	**—**	**—**	**$2,486**	**—**	**—**	**—**	**$2,403**
**15–>37**	185	$4,275	**—**	**—**	**—**	**$3,489**	**—**	**—**	**—**	**$2,674**	**—**	**—**	**—**	**$2,590**
**16–>37**	142	$1,016	**11.13**	**16.14**	**$5,089**	**$0**	**6.28**	**9.11**	**$2,872**	**$0**	**—**	**—**	**—**	**–$8**
**17–>37**	142	$1,033	**—**	**—**	**—**	**$8**	**—**	**—**	**—**	**$8**	**6.28**	**9.11**	**$2,920**	**$0**
**18–>37**	142	$1,089	**—**	**—**	**—**	**$33**	**—**	**—**	**—**	**$33**	**—**	**—**	**—**	**$25**
**19–>37**	185	$3,458	**—**	**—**	**—**	**$2,859**	**—**	**—**	**—**	**$2,044**	**—**	**—**	**—**	**$1,960**
**20–>37**	185	$4,054	**—**	**—**	**—**	**$3,365**	**—**	**—**	**—**	**$2,550**	**—**	**—**	**—**	**$2,467**
**21–>37**	185	$4,275	**—**	**—**	**—**	**$3,553**	**—**	**—**	**—**	**$2,739**	**—**	**—**	**—**	**$2,655**
**22–>37**	185	$3,458	**—**	**—**	**—**	**$2,859**	**—**	**—**	**—**	**$2,044**	**—**	**—**	**—**	**$1,960**
**23–>37**	185	$4,054	**—**	**—**	**—**	**$3,365**	**—**	**—**	**—**	**$2,550**	**—**	**—**	**—**	**$2,467**
**24–>37**	185	$4,275	**—**	**—**	**—**	**$3,553**	**—**	**—**	**—**	**$2,739**	**—**	**—**	**—**	**$2,655**
**25–>37**	142	$1,016	**—**	**—**	**—**	**$23**	**—**	**—**	**—**	**$23**	**—**	**—**	**—**	**$15**
**26–>37**	142	$1,033	**—**	**—**	**—**	**$31**	**—**	**—**	**—**	**$31**	**—**	**—**	**—**	**$23**
**27–>37**	142	$1,089	**—**	**—**	**—**	**$56**	**—**	**—**	**—**	**$56**	**—**	**—**	**—**	**$48**
**28–>37**	248	$1,729	**—**	**—**	**—**	**$1,092**	**2.84**	**7.03**	**$7,255**	**$0**	**—**	**—**	**—**	**–$112**
**29–>37**	248	$1,805	**—**	**—**	**—**	**$1,204**	**—**	**—**	**—**	**$112**	**2.84**	**7.03**	**$7,574**	**$0**
**30–>37**	248	$1,855	**—**	**—**	**—**	**$1,278**	**—**	**—**	**—**	**$186**	**—**	**—**	**—**	**$74**
**31–>37**	248	$1,729	**—**	**—**	**—**	**$1,092**	**—**	**—**	**—**	**$0**	**—**	**—**	**—**	**–$112**
**32–>37**	248	$1,805	**—**	**—**	**—**	**$1,204**	**—**	**—**	**—**	**$112**	**—**	**—**	**—**	**$0**
**33–>37**	248	$1,855	**—**	**—**	**—**	**$1,278**	**—**	**—**	**—**	**$186**	**—**	**—**	**—**	**$74**
**34–>37**	221	$965	**—**	**—**	**—**	**$53**	**—**	**—**	**—**	**$105**	**—**	**—**	**—**	**$101**
**35–>37**	221	$976	**—**	**—**	**—**	**$67**	**—**	**—**	**—**	**$119**	**—**	**—**	**—**	**$114**
**36–>37**	221	$1,013	**—**	**—**	**—**	**$111**	**—**	**—**	**—**	**$163**	**—**	**—**	**—**	**$159**
			**Total cost : model 1**	***$38,591***		**Total cost : model 2**	***$41,499***		**Total cost : model 3**	***$41,866***				

a *There was a supply of 28.5 million calves weighing 0.65 million metric tons at the calf inventory node and demand of 16.14 million metric tons at the packer for all the three scenarios*.

b *The unit for the entry node's cost of production (per metric ton of weight gain), the total cost for beef supply for participating nodes, and the opportunity costs is in million dollars*.

c *The unit for beef flowing out of a node and beef flowing into a node is million metric tons*.

* *The increase in total cost to the model if there is a unit change in the usage of each of the unused nodes is shown as opportunity cost for each of three scenarios*.

#### Possible Network Movements for LHM and HHM Calves

The LHM calves after attaining an acceptable weight can be shifted to a total of 10 different nodes from node 2 (Figure 1). They can move to two types of stocker operations, either an LHM stocker for LHM calves (node 4) or an HHM stocker for LHM calves (node 5), which represented a stocker operation investing in HHM practices (e.g., preconditioning). Similarly, LHM calves from node 2 can move to two types of backgrounder operations, namely, an LHM backgrounder for LHM calves (node 7) or an HHM backgrounder for LHM calves (node 8). Also, some of the LHM calves that have higher weight gain could move directly to six different direct feedlots (nodes 28, 29, 30, 31, 32, 33), classified broadly into two groups based on the quality of the health management (LHM direct feedlots and HHM direct feedlots) practices in these feedlots. The HHM calves after attaining an acceptable weight can be shifted to a total of five different nodes from node 3. HHM calves can move either to a stocker operation (node 6) or to a backgrounder operation (node 9), which strictly implements HHM. To simulate the direct transshipment option to feedlots, under sufficient ADG or acceptable market conditions, the network model had an option for HHM calves from node 3 to move to three different types of HHM direct feedlots (nodes 34, 35, 36).

#### Possible Network Movements for Stockers and Backgrounders

The stockers as well as backgrounders managed under LHM (nodes 4, 7) had the option to move to six different kinds of indirect feedlots (the term “indirect” was used to define feedlots, which received feeder cattle from the intermediate sectors, rather than directly from the calf operations), classified broadly into two types based on the quality of health management. The indirect feedlot groups (nodes 10, 11, 12) were classified as LHM and received LHM stockers. Each of the indirect feedlot (nodes 10 through 27) followed a different health management strategy with respect to ABU (Figure 1). Nodes 13, 14, and 15 were classified as HHM indirect feedlots for LHM stockers and could only raise LHM stockers (Figure 1). Similarly, the indirect feedlots (nodes 19, 20, 21) were classified as LHM and could only receive LHM backgrounders. Nodes 22, 23, 24 were classified as the HHM indirect feedlots for LHM backgrounders and could only raise LHM backgrounders. Thus, stockers and backgrounders raised under LHM facilities (nodes 4, 7) had the option to be fed either in HHM or LHM indirect feedlots. The stockers and backgrounders managed under HHM facilities (nodes 5, 6, 8, 9) could only move to indirect feedlots, which strictly followed HHM. The HHM stockers from nodes 5 and 6 could move to nodes 16, 17, and 18. Likewise, HHM backgrounders from nodes 8 and 9 could move to indirect feedlots with HHM (nodes 25, 26, 27).

#### Possible Network Movements for Feeder Cattle

As explained earlier, both the HHM and LHM feeder calves (with higher ADG) can move to nine different direct feedlots before ending up at the packer (node 37). The feeder cattle from both the HHM and LHM stockers and backgrounders can pass through 18 different indirect feedlots before ending up at the packer. These 27 different feedlots (nine groups of three feedlots) followed one out of the four different health management strategies with respect to ABU as follows:

##### Metaphylaxis and Treatment Feedlots

The first of three feedlots among the six groups (four indirect feedlot groups as well as two direct feedlot groups) that received feeder cattle managed under LHM (Figure 1: nodes 10, 13, 19, 22, 28, 31) followed this ABU strategy. These feedlots received high-risk cattle. They used metaphylaxis upon arrival (20) at the feedlot, and individual feeder cattle with disease were treated for clinical signs of morbidity and mortality (8).

##### Treatment-Only Feedlots

The first of the three feedlots among the three groups (two indirect feedlot groups as well as one direct feedlot groups) that received feeder cattle managed under HHM (Figure 1: nodes 16, 25, 34) followed this ABU strategy. These feedlots received low-risk cattle. These low-risk cattle have low production and mortality risks and are never prescribed metaphylaxis, but are individually treated for clinical signs of morbidity [i.e., pull and treat (8)].

##### ABU-Banned Feedlots

The second of the three feedlots among all the nine groups of feedlots (six indirect feedlot groups as well as three direct feedlot groups) that received feeder cattle managed under both LHM and HHM (Figure 1: nodes 11, 14, 17, 20, 23, 26, 29, 32, 35) followed this ABU strategy. Irrespective of the fact whether these feedlots received high health risk or low health risk feeder cattle, they did not have the option to use ABU either for metaphylaxis or for treatment (26).

##### Grass-Finished Facilities

The third of three feedlots among all the nine groups of feedlots that received feeder cattle managed under both LHM and HHM (Figure 1: nodes 12, 15, 18, 21, 24, 27, 30, 33, 36) followed this ABU strategy. It was assumed that cattle were not confined to feedlots and were finished to slaughter with grazing only, using the acceptable management practices (27). ABU was banned in these facilities also.

This network model can be used to optimize the economic cost to the IBSC under various ABU constraints (e.g., when feedlots are not allowed to use antibiotics in beef production). The optimal lowest cost to produce beef through the supply chain can be obtained by applying a least cost LP optimization model (23) to a linear model representing the costs and weight gain coefficients of beef production for each of the 37 nodes of the network. This then would be the most cost-efficient way to produce beef through the system. Microsoft Excel Solver (28) using an LP specification was used to solve the least cost movement of cattle through the 37 nodes.

### The IBSC Cost of Production Model

To empirically demonstrate how the entire US beef production system could be envisaged as IBSC network flow as described above, we further developed an IBSC cost of production model (Supplementary Tables 1–5). This IBSC cost of production model illustrates how an initial supply of 28.5 million calves [born in year 2018 (10)] is allocated across the consecutive nodes in presence of weight gain and death losses. This allocation enabled us to calculate the four different inputs/constraints required for the LP model, namely,

- The initial supply of beef (in MMT) potentially available from the cow–calf inventory node
- The cost of weight gain (per metric ton) in each of the 37 nodes
- The weight gain coefficient for each of the 37 nodes
- The final weight output from the IBSC network model (in MMT).

Although beef operations are found throughout the United States, many are located in the Great Plains. Thus, individual animal-level information like the initial and final node weights, ADGs, death loss, variable feed, and fixed expenses for each node (Supplementary Tables 1–5) was based on these operations (29, 30), acknowledging that costs and weight gains may differ across regions. The IBSC cost of production model had to be scaled up from the individual-based costs and weight gain coefficients to the herd-level values, in order to calculate the cost of weight gain per MMT and weight gain coefficients of individual nodes in MMT, consistent with the LP model's input requirements. To calculate the herd-level cost of production and weight gain coefficients, the individual animal information was multiplied by the number of animals allocated to each node. Throughout our IBSC cost of production model, we allocated the initial supply of 28.5 million cattle among the 37 different nodes by sequentially splitting the available stock of cattle in an incoming node (Figure 1), equally among all the possible node movements. The number of animals decreases across the nodes because of the mortality. This herd allocation (Supplementary Tables 1–5, first row) enabled the calculation of the different costs per metric ton and weight gain coefficients of each of the 37 nodes for a typical IBSC. This is non-optimal allocation and the optimal allocation are determined by the LP model. Hence, there were 14.25 million each of LHM (node 2) and HHM (node 3) calves (Supplementary Table 2). The 14.25 million LHM calves were split equally among the six possible node movements for node 2. Hence, there were 2.3 million each of stockers, backgrounders, and feeder calves in nodes 4, 5, 7, 8 [one node among the feedlot group (28, 29, 30); one node among the feedlot group (31, 32, 33)], respectively (Supplementary Tables 3, 4). Likewise, the 14.25 million HHM calves were equally split into 4.65 million (Supplementary Tables 3, 4) each between the three possible node movements [node 6, 9 (one among nodes 34, 35, and 36)]. The same logic was used to split the stockers and backgrounders among the indirect feedlot nodes, contributing to 1.12 and 6.81 million feeder cattle in indirect feedlots receiving LHM and HHM animals, respectively (Supplementary Table 5). As standardized units (per metric ton and %) were used, the cost of weight gain per metric ton (Equation 1) and weight gain coefficient (Equation 2) inputs used for the LP scenarios are independent of how animals are allocated across the 37 nodes.

(1) $$\begin{array}{l} Cost\;of\;weight\;gain\;\left(per\;metric\;ton\right)=\\ \;\frac{\left(total\;cost\;of\;production\;for\;the\;node\right)}{\left(final\;herd\;level\;node\;weight-initial\;herd\;level\;node\;weight\right)} \end{array}$$

Hence, the cost per metric ton of weight gain in node 2 (Supplementary Table 2) was the ratio of total cost of production of 13.75 billion to the total weight gain of 3,074,953 metric tons. The total cost of production was the product of per LHM calf rearing cost of $965 and the 14.25 million calves allocated to node 2. The final node weight was 3,398,428 metric tons [product of per weaned LHM calf weight of 238 kg and 14.5 million animals (discounted by 3% death loss)]. The initial herd level node weight, 323,475 metric tons (product of per newborn LHM calf weight of 23 kg and 14.5 million animals), was subtracted from final node weight to obtain total weight gain of 3,074,953 metric ton, for node 2 (Supplementary Table 2).

(2) $$\begin{array}{l} Weight\;gain\;coefficient\;for\;a\;node\;(\%)=\\ \;\left(\frac{final\;herd\;level\;node\;weight\;}{initial\;herd\;level\;node\;weight\;}\right) \end{array}$$

Similarly, for weight gain coefficient for node 2, 1,051% was the ratio of final and initial herd level node weight (i.e., 3,398,428/323,475). The same methodology was followed across the other 36 nodes (Supplementary Tables 1–5) for cost per metric ton and weight gain coefficient calculations. Please refer to footnotes of Supplementary Tables 1–5 for specific details about cost/weight gain coefficient calculations.

#### Cow Inventory Supplying Newborn Calves (Node 1)

This node calculates the cost per metric ton and weight gain coefficient of newborn calves. To simulate approximately 26 million beef cattle slaughtered in the United States annually (10), we started with an initial herd size of 10 million pregnant heifers and 20 million pregnant cows (comparable to the year 2018 US pregnant beef cattle population), which, at 100% pregnancy rate and 95% calf crop, amounted to 28.5 million calves (Supplementary Table 1). The 0.65-MMT weight of 28.5 million calves was used as the supply constraint for the LP scenarios.

#### Cow–Calf Rearing Operations (Nodes 2 and 3)

The cost of production for this sector of the IBSC represents per animal as well as the herd economics of LHM and HHM calf rearing operations (Supplementary Table 2). The newly born calves weighing 23 kg/head from cow–calf inventory were directly transferred to both HHM and LHM cow–calf operations, where they were kept for 245 days until weaning. The ADG of 1.02 kg per day in HHM operations was used vs. 0.91 kg per day in LHM operation to account for better health management, after accounting for the calf's ADG weight distribution reported in the literature (29, 31). We also assumed a lower death rate of 2% in HHM vs. 3% in LHM operation (32). For LHM calves, we used an average calf rearing expense of six different regions of the United States [ADG of 0.91 kg per day (31)]. The expenses in HHM calf operation were slightly higher ($1,018 per head) than in LHM calf operation ($965), considering the higher quality of health management and higher ADG. The breeding cost (Supplementary Table 2) included a cow replacement cost of $140 (29), corresponding to 16% annual replacement rate. The depreciation, taxes, insurance, and opportunity cost of investment are included under other expenses (29).

#### Stockers, Backgrounders, and Direct Feedlots (Nodes 4–9, 28–36)

All the LHM calves in the intermediary nodes had the same initial weight of 238 kg (final weight from node 2), whereas the HHM calves had an initial weight of 267 kg (Supplementary Tables 3, 4). Both LHM and HHM feeder calves were raised for 200 days in all the intermediate nodes. The HHM stockers and backgrounders receiving HHM feeder calves had the highest ADG of 0.91 kg per day and the lowest death loss (2%), when compared to the other four nodes, which received LHM feeder calves (Supplementary Table 3). Just like in cow–calf operations, increased health quality and ADG resulted in increased expenses in HHM nodes (Supplementary Tables 3, 4)s.

Node 28 is an LHM direct feedlot receiving LHM calves. Of the four possible ABU strategies, it uses metaphylaxis and treatment. As antibiotics could be used in feedlot with no limitation, node 28 (also node 31) had the highest ADG (1.6 kg per day, Supplementary Table 4) as well as the second lowest death loss [1.5% (31)] among the direct feedlots. The cost of production model for node 32 (ABU banned feedlot) had slightly lower ADG (1.4 kg per day) and slightly higher death loss (3%, Supplementary Table 4) when compared to node 28. Node 34, which used treatment-only strategy for raising HHM calves, assumed an ADG per day of 1.5 kg and a death loss (30) of 2%. All of the nine direct feedlots raised the feeder calves until they achieved a final slaughter weight of 590 kg. Because of difference in ADG and death rate, the direct feedlot that used metaphylaxis and treatment strategy (nodes 28) had the lowest cost of production per head of $611 (29, 31). The grass-finished facilities (nodes 30, 33, 36) incurred the highest forge cost ($127). The total per-animal expenses were $611, $629, $636, and $655 (Supplementary Table 4), respectively, for direct feedlots using ABU strategies 1, 2, 3, and 4. Only three of nine total direct feedlots are shown in Supplementary Table 4. The calculated costs per metric ton of weight gain and weight gain coefficients of the other six feedlots are available in Table 1.

#### Indirect Feedlots Nodes (Nodes 10–27)

Only 6 of the total of 18 indirect feedlots are shown in Supplementary Table 5. The calculated costs per metric ton of weight gain and weight gain coefficients of all the 18 feedlots are available in Table 1. All the feeder cattle raised in HHM stockers or backgrounders were routed through HHM indirect feedlots. The feeder cattle were kept for different number of days in all feedlots until the final weight of 590 kg (Supplementary Table 5). The same ADGs as well as death losses for the four different ABU strategies used in direct feedlots were used for indirect feedlots. Thus, the lowest ADG (1.10 kg per day) and death loss (0.75%) were for grass-finished facilities, along with the highest total expenses ($563 per head). The highest ADG (1.60 kg per day), as well as the lowest total expenses ($457), was for indirect feedlots using metaphylaxis and treatment strategy. All the other feedlots that used the other two ABU strategies had intermediate ADG and total expenses. The total per-animal expenses were $457, $527, $534, and $563 (Supplementary Table 4), respectively, for indirect feedlots using ABU strategies 1, 2, 3, and 4.

The sum of final weights of 25.8 million fed cattle collated from the nine groups of feedlots (six indirect and three direct), available at the packer node (node 37), was 16.14 MMT (calculation not shown). This 16.14 MMT was used as the demand constraint for the LP models explained below.

### The LP Optimization Model

**Figure 2:**
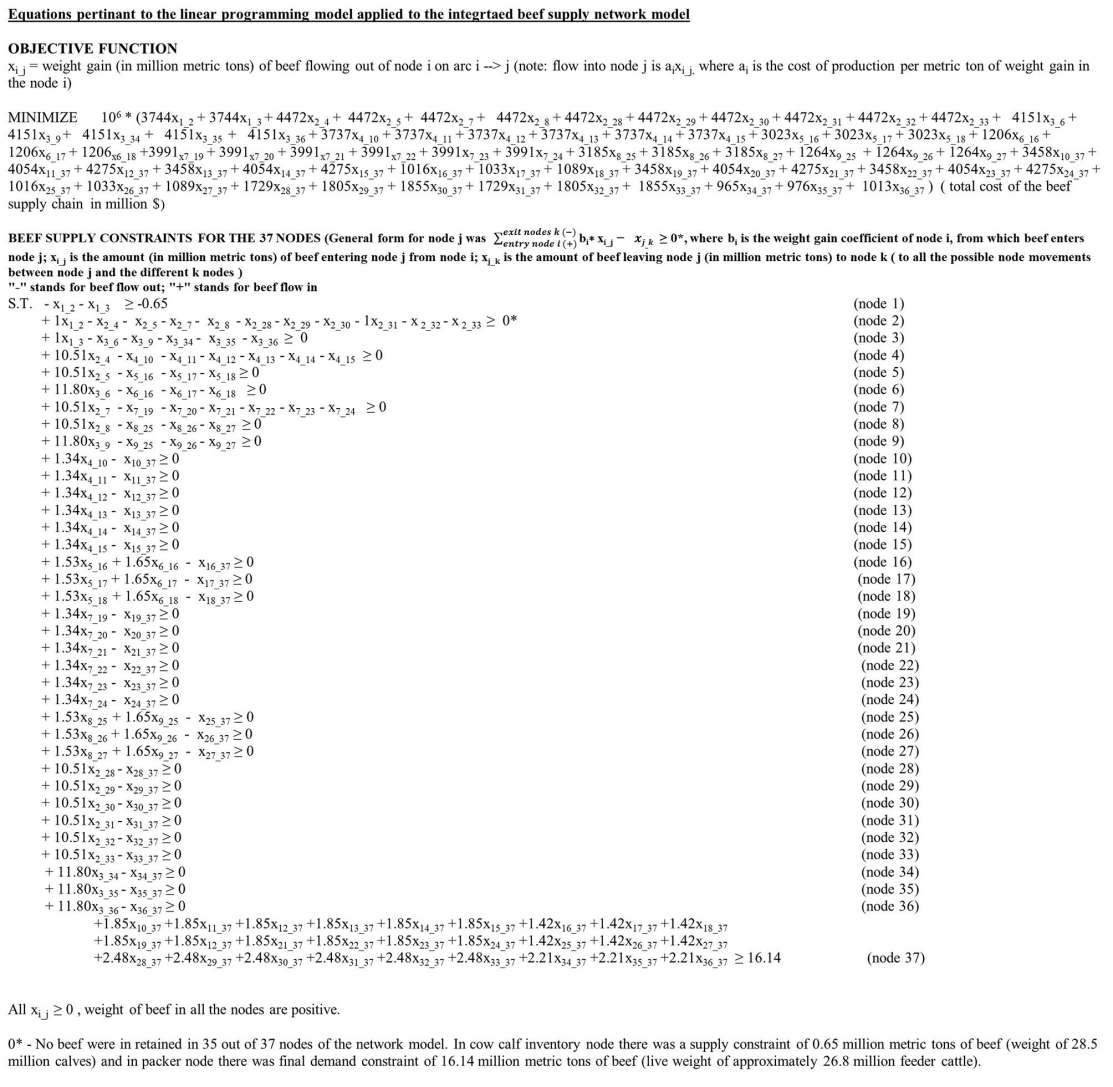
The objective function as well as the 37 different constraint equations for the linear programming model applied to the integrated beef supply chain network model.

Our IBSC network model is analogous to a transportation network model where widgets are transported from point A to a final destination of point Z, with the minimum cost route taken depending on the cost of transportation between nodes, losses of widgets between nodes, and any constraints placed on the throughput through a node. Our model is similar where we determine the least cost movement of cattle through the system based on cost of production and now net weight gain, rather than losses, subject to any constraints on the use of a node. The objective function (Figure 2) that the LP model will minimize is the sum product of the entry node's cost of weight gain for 1 MMT of cattle and the weight gain of cattle (in MMT) using the corresponding node movement, for each of the possible 68 network flows (Table 1). The abbreviated form (see Figure 2 for the full version) of the objective function is shown in Equation 3.

(3) $$\begin{array}{l} Minimize\;10^{6}\;*\;(3,744x_{1\_2}+3,744x_{1\_3}+4,472x_{2\_4}+\ldots \ldots ..\\ \;+\;976x_{35\_37}+1,013x_{36\_37}) \end{array}$$

*where x*_*i*_*j*_ = weight gain (in MMT) of beef cattle flowing out of node *i* on arc *i* –> *j*

The cost of weight gain per MMT for node 1 was the cost of a metric ton of newborn calves ($3,744) × 10^6^. The weight gain in MMT for calves moving from node 1 (cow inventory) to node 2 (LHM calf operation) and node 3 is denoted, respectively, by *x*_1_2_ and *x*_1_3_. The cost of weight gain is the same ($3,744) for variables *x*_1_2_ and *x*_1_3_, as nodes 2 and 3 are receiving the same product (i.e., newborn calves) from the entry node 1. Similarly, $4,472, $976, and $1,013 (Table 1) were the cost of weight gain per metric ton in nodes 2, 35, and 36, for cattle transferred, respectively, to nodes 4, 37, and 37. Likewise, *x*_36_37_ was the total weight gain for fed cattle (in MMT) supplied by the HHM grass-finished facility for HHM calves (node 36) to the packer (node 37).

Each of the 37 nodes had one constraint each, as shown in Figure 2. The general form of constraint equation for any node *j* is denoted by Equation 4.

(4) $$\Sigma_{entry\;node\;i\;\left(+\right)}^{exit\;node\;k\;(-)}\;b_{i}^{*}x_{i\_j}-\;x_{j\_k}\geq 0/\text{any number if mentioned}$$

For all the 37 nodes, “-” sign denotes beef leaving the node *j* and “+” sign denotes beef entering the node *j*. In this constraint equation, *b*_*i*_ denotes the weight gain coefficient of the entry node *i*, from which beef cattle enters node *j*. *x*_*i*_*j*_ is the weight of beef cattle transported between nodes *i* and *j* in MMT. *x*_*j*_*k*_ is the weight (in MMT) of beef cattle exiting node *j* to node *k* (to all possible node movements between node *j* and the different *k* nodes).

For example, the constraint equation of nodes 1 and 3 are denoted by Equations 5, 6.

(5) $$\begin{array}{r} -\;x_{1\_2}-x_{1\_3}\;\geq \;-0.65 \end{array}$$

(6) $$+\;1x_{1\_3}-x_{3\_6}-\;x_{3\_9}-\;x_{3\_34}-\;x_{3\_35}\;-\;x_{3\_36}\;\geq \;0$$

In Equation 5, for the cow–calf inventory, node beef is only exiting the node, and hence, there are no weight gain coefficients. The weights of LHM and HHM calves potentially supplied to nodes 2 and 3 are denoted by –*x*_1_2_ and –*x*_1_3_, respectively. The right-hand side of Equation 5 denotes the 0.65 MMT (weight of the initial supply of 28.5 million calves) leaving node 1. In case of node 3 constraint equation, the weight gain coefficient of the entry node 1 (100%) was used. Beef is entering node 3 from node 1 (x_1___3_). Also, HHM calves after gaining weight have the option to leave the HHM calf operation to HHM stocker (node 6) and HHM backgrounder (node 9), as well as to direct feedlots 34, 35, and 36. All the 35 intermittent nodes (except for nodes 1 and 37) do not retain beef as they are just the weight gaining nodes. Hence, the RHS sides of those 35 constraint equations (Figure 2) are zero. All the fed beef produced in 18 indirect and 9 direct feedlots, weighing 16.14 MMT (calculated from the IBSC cost of production model), enter node 37. Hence, the value 16.14 MMT is used as the demand constraint at node 37 (Figure 2).

The LP model described in Equation 3 was solved with three different ABU constraints to estimate the minimal economic cost to the IBSC network model as shown in Table 1. They are, namely,

#### Scenario 1. Basic Optimization

No constraints to utilize any of the specific production sectors were implemented. The LP model searches through the 68 possible node movements (Table 1), considering the cost of production and weight gain coefficients of these movements, and returns only the node movements required to minimize the total cost of the IBSC network model to meet the demand of 16.14 MMT, using the potential supply of 0.65 MMT of calves.

#### Scenario 2. Calf Supply Constrained

The health status of the newborn calves is equally likely to be low or high health status. Thus, a constraint to supply equal amount of calves to nodes 2 and 3 (i.e., the initial supply of 0.65 MMT was distributed evenly between the calf operations) was implemented in scenario 2. Scenario 2 returns the total least cost to the IBSC network model provided that both high and low health status calves are reared.

#### Scenario 3. ABU Constrained

The purpose of implementing this scenario was to investigate the cost to the IBSC when there was a requirement of using only the antibiotic-free feedlots. Thus, the beef outflow from nine feedlots (nodes 10, 13, 16, 19, 22, 25, 28, 31, 34), which used either metaphylaxis + treatment or treatment-only ABU strategy, were constrained to zero. Scenario 3 then returned the least cost to the IBSC network model to implement ABU-free beef production.

### Sensitivity Analysis

We also investigated two different types of sensitivity analysis for each of these three scenarios, which were implemented using the inbuilt sensitivity analysis package available through Microsoft Excel Solver (28). The sensitivity analysis reports are automatically generated by Microsoft Excel Solver, after calculating the LP solutions. The opportunity cost of an unused node movement (Table 1) is defined as the increase to the total cost of the IBSC network model, if that node is forced into solution by one unit (1 MMT). We also investigated the shadow price of each of the 37 node constraints for the three scenarios (Table 2). The shadow price of any of the node constraint is the increase to the total optimal cost of the IBSC network model, as the RHS of that constraint is increased by 1 unit (1 MMT of beef retention), with all other constraints held fixed.

**Table 2:** The increase in the total cost to the integrated beef supply chain network model when the beef retention in each of the 37 node constraints is incremented by 1 million metric ton (MMT; shadow price), and the allowable range of beef retention (in MMT) for the node is shown for each of the three different linear programming scenarios.

		**Scenario 1. Basic optimization**			**Scenario 2. Calf supply constrained**			**Scenario 3. Antibiotic use constrained**		
		**No constraints on cow–calf, stocker, or feedlot sector**			**Cow–calf inventory has to supply equal amount of calves to both calf operations**			**All indirect and direct feedlots using antibiotics are shut down**		
**Node #**	**Node name**	**Shadow price (million $)**	**Allowable increase (MMT)**	**Allowable decrease (MMT)**	**Shadow price (million $)**	**Allowable increase (MMT)**	**Allowable decrease (MMT)**	**Shadow price (million $)**	**Allowable increase (MMT)**	**Allowable decrease (MMT)**
1	Calf inventory	$0	0.074	1E+30	$2,304	0.05	0.50	$2,868	0.05	0.50
2	LHM calves	$3,744	0.07	0.00	$0	0.05	1E+30	$0	0.05	1E+30
3	HHM calves	$3,744	0.07	0.57	$12,097	0.05	0.25	$13,226	0.05	0.25
4	Stocker	$4,403	0.78	0.00	$4,046	0.56	0.00	$4,046	0.56	0.00
5	Stocker	$4,403	0.78	0.00	$4,046	0.56	0.00	$4,046	0.56	0.00
6	Stocker	$4,116	0.87	6.77	$4,824	0.59	2.95	$4,919	0.59	2.95
7	Backgrounder	$4,403	0.78	0.00	$4,046	0.56	0.00	$4,046	0.56	0.00
8	Backgrounder	$4,403	0.78	0.00	$4,046	0.56	0.00	$4,046	0.56	0.00
9	Backgrounder	$4,116	0.87	0.00	$4,824	0.59	0.00	$4,919	0.59	0.00
10	Feedlot 10	$4,234	1.04	0.00	$3,968	0.75	0.00	$3,968	0.75	0.00
11	Feedlot 11	$4,234	1.04	0.00	$3,968	0.75	0.00	$3,968	0.75	0.00
12	Feedlot 12	$4,234	1.04	0.00	$3,968	0.75	0.00	$3,968	0.75	0.00
13	Feedlot 13	$4,234	1.04	0.00	$3,968	0.75	0.00	$3,968	0.75	0.00
14	Feedlot 14	$4,234	1.04	0.00	$3,968	0.75	0.00	$3,968	0.75	0.00
15	Feedlot 15	$4,234	1.04	0.00	$3,968	0.75	0.00	$3,968	0.75	0.00
16	Feedlot 16	$2,975	1.43	11.13	$3,405	0.96	4.85	$3,463	0.96	0.00
17	Feedlot 17	$2,975	1.43	0.00	$3,405	0.96	0.00	$3,463	0.96	4.85
18	Feedlot 18	$2,975	1.43	0.00	$3,405	0.96	0.00	$3,463	0.96	0.00
19	Feedlot 19	$4,298	1.04	0.00	$4,032	0.75	0.00	$4,032	0.75	0.00
20	Feedlot 20	$4,298	1.04	0.00	$4,032	0.75	0.00	$4,032	0.75	0.00
21	Feedlot 21	$4,298	1.04	0.00	$4,032	0.75	0.00	$4,032	0.75	0.00
22	Feedlot 22	$4,298	1.04	0.00	$4,032	0.75	0.00	$4,032	0.75	0.00
23	Feedlot 23	$4,298	1.04	0.00	$4,032	0.75	0.00	$4,032	0.75	0.00
24	Feedlot 24	$4,298	1.04	0.00	$4,032	0.75	0.00	$4,032	0.75	0.00
25	Feedlot 25	$2,998	1.43	0.00	$3,428	0.96	0.00	$3,486	0.96	0.00
26	Feedlot 26	$2,998	1.43	0.00	$3,428	0.96	0.00	$3,486	0.96	0.00
27	Feedlot 27	$2,998	1.43	0.00	$3,428	0.96	0.00	$3,486	0.96	0.00
28	Feedlot 28	$4,403	0.78	0.00	$4,046	0.56	2.84	$4,046	0.56	0.00
29	Feedlot 29	$4,403	0.78	0.00	$4,046	0.56	0.00	$4,046	0.56	2.84
30	Feedlot 30	$4,403	0.78	0.00	$4,046	0.56	0.00	$4,046	0.56	0.00
31	Feedlot 31	$4,403	0.78	0.00	$4,046	0.56	0.00	$4,046	0.56	0.00
32	Feedlot 32	$4,403	0.78	0.00	$4,046	0.56	0.00	$4,046	0.56	0.00
33	Feedlot 33	$4,403	0.78	0.00	$4,046	0.56	0.00	$4,046	0.56	0.00
34	Feedlot 34	$4,116	0.87	0.00	$4,824	0.59	0.00	$4,919	0.59	0.00
35	Feedlot 35	$4,116	0.87	0.00	$4,824	0.59	0.00	$4,919	0.59	0.00
36	Feedlot 36	$4,116	0.87	0.00	$4,824	0.59	0.00	$4,919	0.59	0.00
37	Packer	$2,367	2.08	16.14	$2,663	1.40	7.03	$2,709	1.40	7.03

## Results

### The IBSC Cost of Production Model

#### Cow–Calf Inventory and Cow–Calf Operations (Nodes 1, 2, 3)

The expenses incurred per head of calf was $85 [(33), Supplementary Table 1], which was assumed to be the cost that the cow–calf inventory incurs to produce the calves. As this cost was assumed to be identical for both HHM and LHM calves, this cost did not influence the optimized results, but was included in the model to obtain the total system cost. For implementing a higher quality of health management, HHM calves incurred a higher per-calf cost of production of $53 ($1,018 to $965). The node cost per ton of weight gain was lower for HHM calves vs. LHM calves ($4,151 vs. $4,472), as a result of higher ADG and lower mortality rate (Supplementary Table 2). Consequently, the HHM calf node weight gain coefficient was higher (1,180%) when compared to that of LHM calves (1,051%).

#### Stocker, Backgrounder, and Direct Feedlot (Nodes 4–9, 28–36)

As the HHM stocker (node 6, $421 per head) and HHM backgrounder ($441 per head in node 9) cost of production was highest among the six intermediary nodes, they had the highest herd expenses of, respectively, $968 million and $1,015 million (Supplementary Table 3). Even though all the stockers as well as backgrounders were kept for 200 days in their respective nodes, the higher ADG and lower death rates in HHM nodes for HHM calves resulted in higher final node weight per head of 440 kg (Supplementary Table 3) before being sent to indirect feedlots. Hence, the node cost per metric ton of weight gain was lowest for these HHM nodes ($1,206 and $1,264, Supplementary Table 3). The higher ADG also contributed to higher weight gain coefficients for HHM nodes raising HHM calves when compared to the other four stocker/backgrounding nodes in Supplementary Table 3.

In case of direct feedlots, to attain a final slaughter weight of 590 kg, the LHM and HHM feeder calves had to be kept in feedlots for days ranging from 227 to 288 (Supplementary Table 4). As the total expenses were lowest ($611 per head) in feedlots using metaphylaxis and treatment, they had the lowest cost per ton of weight gain among a specific feedlot group raising a specific type of feeder calf. In Table 1, if one compares the cost and weight gain coefficients for node movements from nodes 28 through 36 to packer node 37, nodes 28, 31 ($1,729), and 34 ($965) had the lowest cost per metric ton of weight gain. The HHM direct feedlots for HHM calves (nodes 34, 35, 36) had smaller weight gain coefficients due to the higher initial weights in these nodes (Supplementary Table 4).

#### Indirect Feedlots (Nodes 10–27)

The HHM indirect feedlots receiving HHM stockers and backgrounders (nodes 16–18, 25–27, Supplementary Table 5) had a higher feeder weight of 415 kg compared to 318 kg in the other 12 nodes receiving LHM stockers, as well as backgrounders. The cost of production was higher in grass-grazed facility ($563, Supplementary Table 5) vs. feedlots that used metaphylaxis and treatment ($457 in nodes 10 and 19, Supplementary Table 5). Hence, the feedlots using metaphylaxis and treatment strategy had the lowest cost per ton of weight gain among a specific feedlot group raising a specific type of feeder cattle. In Table 1, if one compares the cost and weight gain coefficients for node movements from nodes 10 through 27 to packer node 37, for LHM indirect feedlots receiving LHM stockers and backgrounders, the nodes 10, 13, 19, and 22 had the lowest cost per metric ton of weight gain ($3,458). The weight gain coefficients for HHM indirect feedlots raising HHM feeder cattle (nodes 16–18, 25–27) were lower (145%) because of the higher initial weights in these nodes (Supplementary Tables 5, 1).

### The LP Optimization Scenarios

#### Scenario 1. the Basic Optimization Model

**Figure 3:**
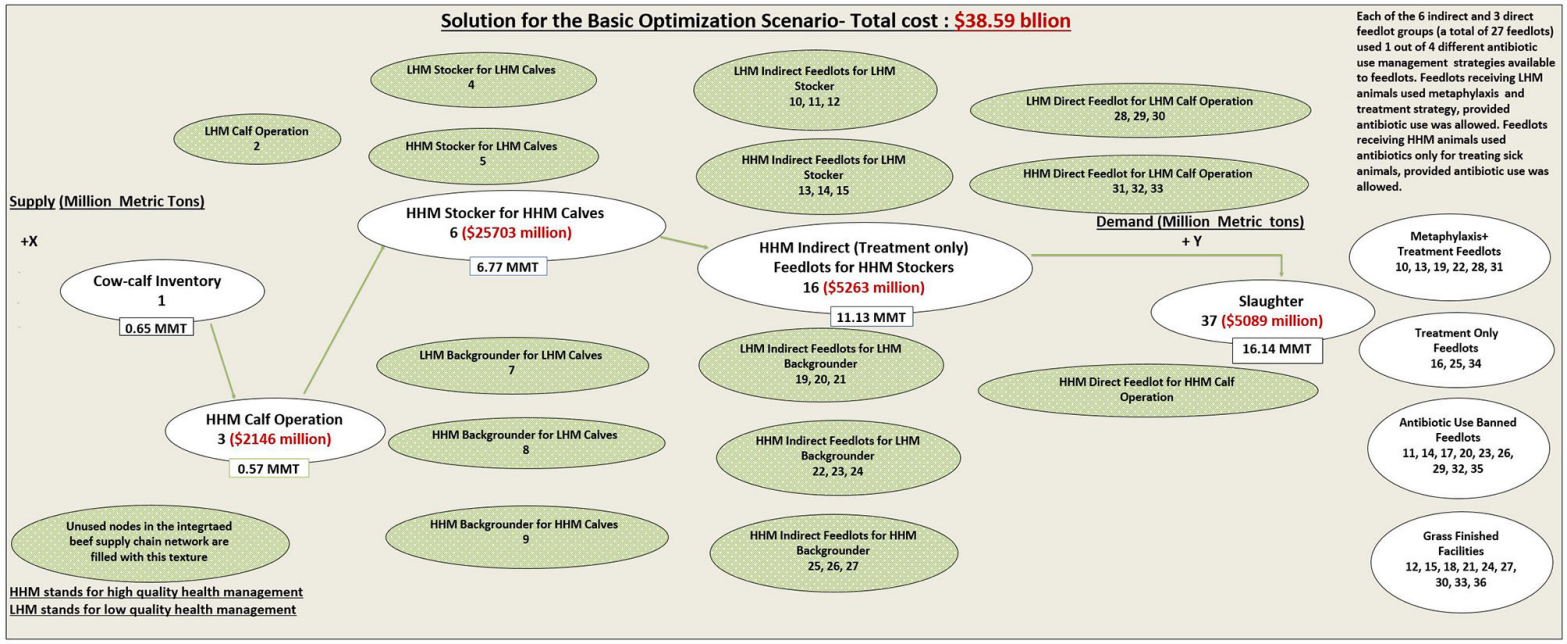
The schematic representation of the optimal solution for the basic optimization scenario with the 5 used nodes through which the least cost beef supply happens is shown.

Scenario 1 required only four node movements (13, 36, 6 16, 1637, Table 1) to meet the final demand of 16.14 MMT, at least cost. To visualize the solution in the framework of our IBSC network model, the solution for scenario 1, with the associated 4 optimal node movements, is also depicted in Figure 3. This scenario required only a supply of 0.57 MMT (out of a total available supply of 0.65 MMT) of the HHM calves from the cow–calf inventory, at a cost of $2,146 million (weight gain of 0.57 MMT ^*^ per metric ton cost of production of calves of $3,744 million). The LHM calves did not enter the solution given their higher total costs through the system, implying that HHM calves are more cost-efficient than LHM calves for the system. The 0.57 MMT of HHM calves gained weight for 240 days in HHM calf operation (node movement 36) to become weaned calves weighing a total of 6.77 MMT (0.57 ^*^ weight gain coefficient of 1,180%, Table 1) at a weight gain cost of $25,703 million (weight gain of 6.2 MMT ^*^ node cost of $4,151 million). Then, the weaned calves moved to node 6 (HHM stocker for HHM calves). The 6.77 MMT of weaned calves increased in weight (at weight gain coefficient of 165%) to become 11.13 MMT weighing HHM stockers at a weight gain cost of $5,263 million and moved to indirect feedlot 16 (treatment-only feedlot for HHM stockers). In feedlot 16 (node movement 16 37), the HHM stockers increased in weight to a per-head weight of 590 kg to meet the final demand of 16.14 MMT and are available at node 37 for slaughter. The cost incurred by node 16 for weight gain from 11.13 MMT to 16.14 MMT was $5,089 million, resulting in a total cost to the IBSC network model of $38.59 billion (Figure 3 and Table 1).

#### Scenario 2. Calf Supply Constrained

Scenario 1 only sourced HHM calves for meeting the final demand as it was cheaper to only use HHM calves. However, if LHM calves are produced, then these calves must be used in the beef production system. As a result of the constraint implemented, scenario 2 sourced both LHM and HHM calves weighing 0.32 MMT each, at a cost of $1,211 million (Table 1). Both the LHM and HHM calves gained weight for 240 days, resulting in weaned calves weighing a total of 2.84 and 3.82 MMT in nodes 2 and 3, respectively. LHM calves weighing 0.05 MMT were retained in node 2 (node movement 228 only used 0.27 MMT, Table 1) as they were not required to meet the final demand of 16.14 MMT, subject to the minimum cost constraint for LP 2. The feeder calves from node 2 moved to LHM direct feedlot with metaphylaxis and treatment for LHM calf operations (node movement 228, Table 1), at a cost of $11,472 million for the weight gain from 0.27 MMT to 2.84 MMT. In feedlot 28, the 2.84 MMT of LHM feeder calves achieved a final weight of 7.03 MMT at a cost of $7,255 million and was moved to packer (node movement 2837). The weaned calves from node 3 were moved to HHM stocker for HHM calf operation (node movement 36), at a weight gain cost of $14,507 million. The weaned HHM calves weighing 3.82 MMT gained weight to stockers weighing 6.82 MMT at $2,971 million (node movement 616). In feedlot 16, the HHM stockers gained weight, reaching a final weight of 9.11 MMT, at a cost of $2,872 million, thereby meeting the final demand of 16.14 MMT together with node 28 contributing to 7.03 MMT of fed cattle. The total cost incurred by scenario 2, utilizing the seven node movements shown, was $41.5 billion, an increase of $2.9 billion when compared to scenario 1. Thus, $2.9 billion is the cost of having LHM calves in the beef system as compared to only HLM calves.

#### Scenario 3. ABU Constrained

Scenario 2 used nodes 16 and 28, which used antibiotics for feeder cattle management. Scenario 3 estimated the minimum cost for the IBSC network model for using only antibiotic-free feedlots, by restricting the use of nine feedlots, which allowed ABU in feeder cattle management. Again, there was a constraint to use both the LHM and HHM calf operations equally. Hence, 0.32 MMT of LHM and HHM calves were sourced by nodes 2 and 3 (Table 1). Only 0.27 MMT of LHM calves from node 2 were required out of the supply of 0.32 MMT to supply feeder calves weighing 2.84 MMT to node 29. Meanwhile, node 3 utilized all the 0.32 MMT of HHM calves to supply a higher quantity of stocker weighing 3.82 MMT (node movement 36). As beef flows out from nodes 16 and 28 were constrained to 0, the feeder calves from nodes 2 and 3 were routed through nodes 17 and 29, respectively (the feedlots with the next higher node cost of $1,805 and $1,033, respectively, Table 1). Scenario 3 incurred a total cost of $41,866 million, an increment of $367 million over scenario 2 cost. Thus, $367 million is the additional minimum cost for restricting feedlots using antibiotics in our IBSC network model.

### Opportunity Cost

If a production node does not enter the LP solution, then it is not optimal to use that node to arrive at the least system cost of beef production. The increase in system cost that results by forcing a node into solution represents the opportunity cost of using that node. To calculate the opportunity cost, the model simulates alternative routes that result in a particular node movement within the supply chain (compared to the optimal least-cost route), after considering the cost and weight gain coefficients of upstream and downstream node movements that is causing or resulting from the node movement for which opportunity cost is calculated. The opportunity cost in model 1 for using node movement (5→ 16, Table 1) was $1,454 million, the additional cost incurred by the IBSC network model for channelizing 1 MMT of beef by utilizing this node movement. To use this node movement (5→ 16), a specific amount of LHM calves will have to be sourced from cow inventory, and node 2 should supply the weaned calves to node 5, before they reach node 16 and subsequently node 37 (Table 1, see the possible network flows ending up in node 16). So the inbuilt sensitivity analysis Excel package calculates opportunity costs of relevant nodes for each scenario. The LP model uses the combined lower weight gain coefficients and higher costs of nodes 2 and 5 when compared to the combined higher weight gain coefficient/lower costs of nodes 3 and 6 to calculate this higher cost of $1,454 million. The same node movement logic can explain the opportunity costs for all the other feedlots for scenario 1. The concept of opportunity cost for node movement 28→ 37 (scenario 2) vs. 29→ 37 (scenario 3) is more straightforward. When the 2.84 MMT of feeder cattle were fed in node 29 (in scenario 3), the system cost was $112 million higher than the 2.84 MMT of cattle fed in node 28 for scenario 2 (see the opportunity costs for node movements 28→ 37 and 29→ 37 for scenarios 2 and 3, Table 1). In case of scenario 3, node movement 28 (metaphylaxis and treatment feedlot)→ 37 was avoided and 29 (ABU banned feedlot)→ 37 as the scenario avoided feedlots using antibiotics.

### Shadow Price

An LP model is a constrained optimization problem, and the constraint equations have Lagrangian values or shadow prices (28), which is the change in the objective value if a constraint is relaxed by one unit, which in our case is an MMT of beef retention in 1 of the 37 nodes of the IBSC network model. In contrast to the opportunity cost, where a possible node movement is forced into solution by one unit, the shadow price reflects the incremental change in the total system cost, when a constraint is relaxed by one unit of production. In case of scenario 1, the shadow price of node 1 was $0 (Table 2), as no additional cost is incurred to retain some calves not needed for beef production, up to an allowable increase of 0.074 MMT. Hence, in LP scenario 1 (Table 1), only 0.57 MMT of calves were sourced from cow–calf inventory. The shadow price for nodes 2 and 3 was $3,744 million each for retaining calves weighing 1 MMT, valid only up to the allowable increase range of 0.07 MMT (Table 2), outside of which the optimal solution for the IBSC network model will change. In case of scenarios 2 and 3, LHM calves weighing 0.05 MMT (Tables 1, 2) were retained in node 2 to reduce cost, at a shadow price of $0. Had the scenario solution retained HHM calves in node 3, scenarios 2 and 3 would have incurred a higher cost of, respectively, $12,097 million and $13,226 million for increasing the HHM calves retention by 1 MMT. In case of node 37, scenario 1 could have supplied for up to 2.08 MMT (Table 2, node 37's allowable increase) more of fed beef over the current supply of 16.14 MMT utilizing other costly node combinations. However, it will incur an additional cost of $2,367 million cost for every 1-MMT supply. The shadow prices for a specific feedlot group with the same efficiency (for all the nine groups of feedlots) were similar, across all the three LP scenarios.

## Discussion

### The Utility of IBSC Network Model

The objective of our study was to estimate the economic cost to the US beef system for various plausible ABU restrictions. Economic estimates of using ABU reduction technologies (34) will aid US beef industry in implementing policies leading to overall reduction in ABU. Recently, there has been a surge of interest in the IBSC in US beef production (12, 35). In the current big data age, traceability systems specific to IBSC (14) can be utilized to capture the health status of individual animals (36) throughout the supply chain. These collected data can be utilized to make decisions concerning the key profitability or sustainability variables such as the potential for AMR transmission to humans. Quite often in disintegrated beef supply chains, each sector will focus on management decisions that can maximize the individual sector profit, which can adversely affect the profit of the subsequent sector. By arriving at the minimum cost of producing beef through the whole beef production system, gains to the entire sector (37) and the economy are maximized. In an efficient market, these gains would be equitably distributed (38) over the various sectors of the system.

Our IBSC network model assumed that the producers can differentiate between LHM and HHM animals upon arrival at a production node. The general rule of thumb followed across the IBSC network model was that the HHM animals will have higher ADG, lower mortality rates, and hence lower cost of production and higher weight gain coefficients. The separation of nodes into HHM and LHM was conceptual and was introduced to model the fact that the health management of animals is one of the major contributing factors for the difference in the range of ADG as well as mortalities (39) observed in beef systems. Differential routing of animals based on their health status as shown in our network model can minimize the comingling (40) of LHM and HHM animals, one of the significant disease transmission–enabling factors for BRD.

### The Utility of LP Optimization Model

As our LP scenarios minimized the total cost of the IBSC network model, the LP scenarios 1 and 2 used node movements involving feedlots (nodes 16, 28, Table 1) using metaphylaxis and treatment strategy (vs. feedlots using other ABU strategies) because of their lower costs. The reason why scenario 1 only used HHM calves for meeting the final demand is also the lower node cost ($4,151 vs. $4,472, Table 1) and higher efficiency of node 3 vs. node 2. When a constraint of utilizing equal proportions of HHM and LHM calves was forced in scenario 2, the node movements (2 28 and 2837) entered solution to use the LHM calves in the production process. Utilizing equal proportion of LHM and HHM increased the cost of scenario 2 by 7.5% when compared to scenario 1 cost of $38,591 million (Table 1). This implies that significant cost reductions could be accomplished in the beef sector model if only high health calves were produced. In scenario 3 when the outflows from the nine feedlots that used antibiotics were constrained to 0, the total cost to the network model increased by $367 to $41,866 million, which is a 0.90% increase in cost when compared to scenario 2 cost of $41,499 million. Our estimate for the cost of banning ABU in US feedlot ($367 million) is dependent on the relative differences in node cost per MMT of weight gain in direct and indirect feedlots using the four different ABU strategies. Our estimate of 0.90% increase in cost of production for banning ABU from beef production is similar to the 1% to 3% increase in cost of production that Sneeringer et al. (34) found when investigating the economics of ABU ban in all the livestock species. Recently, Dennis et al. (8) estimated the value of metaphylaxis as $532 million (0.92% of industry gross revenue) when conducting a cost–benefit analysis between feedlots using metaphylaxis vs. no metaphylaxis. Also, Olvera (41) using a whole system structural econometric model, estimated 1% reduction in US beef production, following the ban of feed-grade antibiotics.

What we have modeled is a simplified US beef production system that comprises 730,000 cow–calf operators, 230,000 stocker/backgrounders, and 75,000 feedlots, each with their own inherent cost of productions. The result is that the LP solutions presented here can only give a glimpse into the impact of restricting ABU among those myriad of producers. The LP model can be applied for other combinations of cost of productions, supply of calves, final demand, and weight gain coefficients. A potential impact of this diversity of operations with various costs and efficiencies can be gauged by the opportunity costs that would be incurred if a node was forced into the LP solution. One of the limitations of our model is the lack of accounting of uniform prevalences of the most common bovine infectious and production diseases such as BRD, liver abscess, or lameness, for which antibiotics are used in beef production. While the disease prevalences vary across different production practices, the reported mean prevalences of BRD, liver abscess, and lameness in North American beef production are, respectively, 16 (42), 12–34% (43), and 32% (44). Ideally, having cost of production parameters from a controlled experiment at the same prevalence of these diseases in each of different feedlots using the four different ABU strategies should be used to obtain more precise estimation of the value of antibiotics in beef systems. Yet another economic externality that we have not accounted for is the potential change in beef retail prices, as a result of reduction in production by switching a significant portion of conventional beef systems to reduced/ABU banned beef systems. This expected change in commodity as denoted by elasticity is extensively discussed by Dennis et al. (8).

A sustainable beef system with its economic-, societal-, and environmental sustainability–related goals should include a variety of constraints before the true value of antibiotics to the US beef system can be determined. To consider stocker/backgrounder sector participation in beef production, the opportunity costs for HHM stockers and backgrounders presented in Table 1 show the additional cost that the IBSC network model will incur when either the HHM stocker or backgrounder is forced into the model solution to accommodate the intermediate sector in beef production. Depending on the availability of forage and feedlot capacity, the beef system has a seasonal nature, and consequently, several of the calves are redirected through the stocking or backgrounding sectors, until there is feedlot availability (an economic resource constraint). In LP scenario 1, 0.074 MMT of calves (~3 million calves) were not utilized (Table 1), as only 0.57 MMT of calves were required for meeting the final demand. So given the supply, demand, and constraints of the IBSC, one can limit the supply (calves required) for the subsequent year's production cycle. The shadow price generated as part of sensitivity analysis of the IBSC network model shows the additional cost of retaining additional 1 MMT of animals in any of the 37 nodes of production to be in harmony with the production cycle or resource constraints.

Using an integrated systems approach, we estimated the minimal cost of banning ABU (an environmental goal) in beef production, to aid beef industry's effort in contributing to the global efforts (45) mitigating AMR. However, the sustainable optimum with regard to ABU for the beef systems is not a complete ban. Given the fact that there will still be incidence of prevalent bovine diseases, even after improving the known disease mitigation and surveillance efforts, treatment using antibiotics is a requirement for maintaining acceptable animal welfare (46) standards (a societal goal). Even if ABU in beef production cycle is minimized to just maintain the acceptable animal welfare standards, the different antibacterial interventions implemented during slaughter and fabrication processing can reduce bacterial loads significantly (47) in retail meat samples, thereby maintaining acceptable food safety standards. However, the routine AMR surveillance done by the US national AMR monitoring program has reported that more than 50% of samples containing the common foodborne pathogens such as *Campylobacter* (48) and *Salmonella* (49) exhibited multidrug resistance, even after the routine antibacterial interventions implemented during slaughter. Hence, the optimal ABU in beef systems should be arrived at by considering all the societal, environmental, and economic goals in consultation with all the stakeholders (50). The spread of AMR bacteria and genes across human and animal health systems can occur mainly through wastewater, soils, manure applications, direct exchange between humans and animals, and food exposure (51). Differences in health practices followed on farm, microbial genetics, and resistance accrual mechanisms, as well as social and human factors, make the tracing of origin and drivers of AMR bacteria and genes arising from beef systems an uphill task. Once AMR transmission risk parameters corresponding to each of four ABU strategies we have in the model become available from routine antimicrobial surveillance, we could further modify our model to include corresponding AMR transmission risks for each of the 27 feedlot nodes in our model, which is currently not available at the level necessary for such a model. The inclusion of this risk as a constraint will obviously provide a different solution than the solution we have elucidated in Table 1. Such type of multiobjective optimization problems (23) can be evaluated by the IBSC network model used for this study with further modifications.

## Conclusions

There is increasing demand from consumers for beef free of antibiotic residues. This can be accomplished by shifting beef production from conventional production systems that use antibiotics for both treatment and prevention, to production systems implementing strict antimicrobial stewardship practices. Alternative production practices usually have higher production costs due to lower weight gain efficiency and higher disease costs. The diverse production practices, as well as differing production costs of the US beef supply chain, poise a challenge to estimate the costs of beef production under various ABU management programs. A generalizable conceptual IBSC network model was constructed, and LP optimization was applied to this network model to determine the beef production practices that minimized the cost of the IBSC. The economic costs incurred by the network model under three different ABU constraints were estimated. The increase in production cost for shifting from the conventional feedlot practices, utilizing antibiotics to antibiotic-free production systems, depends on the relative cost of beef production, as well as weight gain efficiencies of the different ABU management strategies. Our estimate of an increase in cost of $367 million for implementing antibiotic-free production systems was only 0.90% of the total minimum cost of $41,499 million incurred for implementing the conventional beef production. However, any policy that limits the usage of antibiotics must also consider the animal welfare and food safety risks associated with beef production.

## Data Availability Statement

The original contributions presented in the study are included in the article/Supplementary Material, further inquiries can be directed to the corresponding authors.

## Author Contributions

LWT and YTG conceptualized the study, obtained funding, designed the experiments and wrote the article. KK build the model, designed the experiments, and wrote the article. All authors contributed to the article and approved the submitted version.

## Conflict of Interest

The authors declare that the research was conducted in the absence of any commercial or financial relationships that could be construed as a potential conflict of interest.
