# Supplementary material for: System Economic Costs of Antibiotic Use Elimination in the US Beef Supply Chain
Source: Front Vet Sci. 2021 Apr 26;8:606810. doi: 10.3389/fvets.2021.606810 (PMC8107467; doi:10.3389/fvets.2021.606810)
Supplement: Supplementary file 1 [file Data_Sheet_1.docx]

**11 Supplementary Materials**

**Table S1.** The cost of production for the cow-calf inventory calculated on per calf and the whole herd basis.

|  | | **Cow calf inventory (node 1)** | |
| --- | --- | --- | --- |
|  | Per calf | | Herd |
| Number of heifers | 1 | | 10,000,000 |
| Number of cows | 2 | | 20,000,000 |
| Heifer Alive through pregnancy (%) | 100% | | 100% |
| Cows Alive through pregnancy (%) | 100% | | 100% |
| Calf crop from heifers (%) | 95% | | 95% |
| Calf crop from cows (%) | 95% | | 95% |
| Total number of calves available | 3 | | 28,500,000 |
| Calf weight at birth (kg/metric tons) | 23 | | 646,950 |
| Cost of calf at birth ($ per calf/per herd) | $85 | | $2,422,500,000 |
|  |  | |  |
| **Node cost^#^ (per metric ton of weight)** | **-** | | **$3,744** |
| **Weight gain coefficient*** | **100%** | | **100%** |

#Node cost per metric ton of calf weight is the herd cost ($2.4 billion) to total weight (646,950) ratio of 28.5 million calves

*Weight gain coefficient 100% is the ratio of herd final weight (646,950) and herd initial weight (646,950); 100%. There is no weight gain in calf inventory

**Table S2.** The cost of production for the calf operations (both low and high quality health management: nodes 2 and 3) calculated on per animal and herd basis

| Number of calves allocated | 14,250,000 | | 14,250,000 | |
| --- | --- | --- | --- | --- |
|  | **Low quality health management (node 2)** | | **High quality health management (node 3)** | |
|  | Per animal | Herd | Per animal | Herd |
| Calf weight (kg/metric tons) | 23 | 323,475 | 23 | 323,475 |
| Days in phase (days) | 245 | 245 | 245 | 245 |
| ADG per day (kg) | ***0.91*** | ***0.91*** | ***1.02*** | ***1.02*** |
| Death loss (%) | ***3%*** | ***3%*** | ***2%*** | ***2%*** |
| Final node weight (kg/metric tons) | 238 | 3, 398,428 | 267 | 3,818,461 |
|  |  |  |  |  |
| **Cow calf expenses** |  |  |  |  |
| Vet/vaccine/drugs | $27 | $384,750,000 | $32 | $456,000,000 |
| Labor | $50 | $712,500,000 | $58 | $826,500,000 |
| Breeding | $174 | $2,479,500,000 | $180 | $2,565,000,000 |
| Marketing | $24 | $342,000,000 | $27 | $384,750,000 |
| Utilities/Machinery | $74 | $1,049,750,000 | $77 | $1,092,500,000 |
| Interest on operating/other | $222 | $3,165,875,000 | $225 | $3,208,625,000 |
| **Feed expenses** |  |  |  |  |
| Pasture/crop residue | $203 | $2,907,000,000 | $219 | $3,120,750,000 |
| Harvested forage | $121 | $1,729,000,000 | $126 | $1,800,250,000 |
| Supplements/Minerals | $69 | $980,875,000 | $74 | $1,052,125,000 |
| Total cost of production | $965 | $13,751,250,000 | $1,018 | $14,506,500,000 |
|  |  |  |  |  |
| **Node cost^#^ (per ton of weight gain)** | ***-*** | **$4,472** | ***-*** | **$4,151** |
| **Weight gain coefficient*** | ***-*** | ***1051%*** | ***-*** | ***1180%*** |

#Node cost per metric ton of LHM calf (node 2) weight gain is the ratio of total cost of production ($13.75 billion: node 2) to weight gain (3074953 metric ton) of 14.25 million calves. Same logic is used in case of node 3 as well.

*Weight gain coefficient 1051% of LHM calves is the ratio of total final weight (3,398,428) and the total initial weight at node (323,475). Same logic is used in case of node 3 as well

**Table S3.** The cost of production model for the 3 stocker and the 3 backgrounder operations (both low health as well as health quality management) calculated per animal basis.

| Number of cattle allocated | 2,303,750 | 2,303,750 | 4,655,000 | 2,303,750 | 2,303,750 | 4,655,000 |
| --- | --- | --- | --- | --- | --- | --- |
|  | **LHM Stocker for LHM Calves**  **(node 4)** | **HHM Stocker for LHM Calves (node 5)** | **HHM Stocker for HHM Calves (node 6)** | **LHM Backgrounder for LHM Calves (node 7)** | **HHM Backgrounder for LHM Calves (node 8)** | **HHM Backgrounder for HHM Calves (node 9)** |
|  | Per animal basis | | | | | |
| Stocker/backgrounder weight (kg) | 238 | 238 | 267 | 238 | 238 | 267 |
| Days in phase (days) | 200 | 200 | 200 | 200 | 200 | 200 |
| ADG per day (kg) | ***0.45*** | ***0.68*** | ***0.91*** | ***0.45*** | ***0.68*** | ***0.91*** |
| Death loss (%) | 3.0% | 2.5% | 2.0% | 3.0% | 2.5% | 2.0% |
| Final node weight (kg) | 318 | 364 | 440 | 318 | 364 | 440 |
|  |  |  |  |  |  |  |
| **Stocker/backgrounder expenses** |  |  |  |  |  |  |
| Vet/vaccine/drugs | $10 | $24 | $30 | $15 | $29 | $35 |
| Labor | $18 | $38 | $43 | $27 | $47 | $52 |
| Marketing | $5 | $10 | $15 | $8 | $13 | $18 |
| Fuel and energy | $10 | $17 | $24 | $12 | $19 | $26 |
| Machinery | $2 | $6 | $8 | $4 | $8 | $10 |
| Interest on cattle loan/other | $8 | $12 | $15 | $11 | $15 | $18 |
| **Feed Expense** |  |  |  |  |  |  |
| Wintering/Backgrounding cost | $120 | $135 | $140 | $155 | $170 | $175 |
| Summer grazing | $120 | $130 | $132 | $72 | $82 | $84 |
| Mineral and salt | $10 | $12 | $14 | $19 | $21 | $23 |
| Total cost of production | ***$303*** | ***$384*** | ***$421*** | ***$323*** | ***$404*** | ***$441*** |
|  |  |  |  |  |  |  |
| **Herd level variables**  Total cost of production for node | $696,884,375 | $883,488,125 | $968,726,875 | $744,111,250 | $930,715,000 | $1,015,953,750 |
| Total initial weight (metric ton) | 547,844 | 547,844 | 1,245,250 | 547,844 | 547,844 | 1,245,250 |
| Total final weight (metric ton) | 734,314 | 840,074 | 2,048,786 | 734,314 | 840,074 | 2,048,786 |
| **Node cost^#^ (per ton of weight gain)** | **$3,737** | **$3,023** | **$1,206** | **$3,991** | **$3,185** | **$1,264** |
| **Weight gain coefficient*** | **134%** | **153%** | **165%** | **134%** | **153%** | **165%** |

#Node cost per metric ton of weight gain for the node 4 stockers is the ratio of total node cost of production ($696 million) to weight gain (186470 metric ton) of 2.30 million stockers. Same logic used in case of nodes 5, 6, 7, 8, and 9

*Weight gain coefficient 134% of node 4 stockers is the ratio of total final weight (734,314) and the total initial weight at node (547,844). Same logic used in case of nodes 5, 6, 7, 8, and 9

**Table S4.** The cost of production for the 3 groups* of direct feedlot operations which received feeder calves directly from both the low health as well as high health quality managed calf operations

| Number of cattle allocated | 2,303,750 | 2,303,750 | 4,655,000 |
| --- | --- | --- | --- |
|  | **LHM direct feedlot for LHM Calves using metaphylaxis and treatment strategy (node 28)** | **HHM direct feedlot for LHM Calves using ABU Banned strategy (node 32)** | **HHM direct facility for HHM Calves using treatment only strategy (node 34)** |
|  | Per animal basis | | |
| Feeder weight (kg) | 238 | 238 | 267 |
| Days in phase (days) | 227 | 272 | 227 |
| ADG per day (kg) | ***1.6*** | ***1.4*** | ***1.5*** |
| Death loss during feeder phase (%) | ***1.5%*** | ***3.0%*** | ***2.00%*** |
| Final node weight (kg) | 590 | 590 | 590 |
|  |  |  |  |
| **Feedlot expenses (per head)** |  |  |  |
| Vet/vaccine/drugs | $17 | $11 | $15 |
| Labor | $29 | $29 | $30 |
| Marketing | $19 | $21 | $20 |
| Fuel and energy | $24 | $27 | $24 |
| Machinery | $22 | $23 | $22 |
| Interest on cattle loan/Other | $37 | $31 | $35 |
| Fixed expense | $49 | $115 | $87 |
| **Feed Expense** |  |  |  |
| Harvested forage cost | $30 | $28 | $29 |
| Grain/supplement | $351 | $322 | $337 |
| Mineral | $32 | $29 | $31 |
| Total expenses during the phase | ***$611*** | ***$636*** | ***$629*** |
| **Herd level variables** |  |  |  |
| Total cost of production for node | $1,406,909,559 | $1,464,403,855 | $1,450,226,713 |
| Total initial weight (metric ton) | 547,844 | 547,844 | 1,245,250 |
| Total final weight (metric ton) | 1,351,237 | 1,359,261 | 2,748,301 |
| **Node cost^1^ (per ton of weight gain)** | **$1,729** | **$1,805** | **$965** |
| **Weight gain coefficient^2^** | **248%** | **248%** | **221%** |

*All the 3 groups had 3 separate feedlots (so a total of 9 direct feedlots^#^), each of which followed a specific antibiotic use strategy out of the 4 possible antibiotic use strategies, namely 1) Metaphylaxis along with treatment of sick animals (Only feedlots receiving low quality health feeder cattle could use this strategy), 2) Treatment of sick animals only (only feedlots receiving high quality health feeder cattle could use this strategy), 3) Feedlots in which any use of antibiotics was banned, 4) Feeder cattle are exclusively grass-grazing and antibiotic use was banned

#The other 6 out of a total of 9 different (nodes 29, 30, 32-25) node costs and weight gain coefficients for direct feedlots used in the optimization models are available in table 1

^1^Node cost per metric ton of weight gain for the node 28 feeder calves is the ratio of total node cost of production ($1.406 billion) to weight gain (803,393 metric ton) of 2.30 million feeder calves. Same logic used in case of nodes 29-36.

^2^Weight gain coefficient 248% of node 28 is the ratio of total final weight (1, 351,237) and the total initial weight at node (547,844). Same logic used in case of nodes 29-36

**Table S5.** The cost of production for the 6 groups* of indirect feedlot operations which received feeder cattle from stocker as well as backgrounder operations

| Number of cattle allocated | 1,117,319 | 1,117,319 | 6,808,056 | 1,117,319 | 1,117,319 | 6,808,056 |
| --- | --- | --- | --- | --- | --- | --- |
|  | **LHM indirect feedlot for LHM stockers using strategy* 1 (node 10)** | **HHM indirect feedlot for LHM stockers using strategy* 3 (node 14)** | **HHM indirect feedlot for HHM stockers using strategy* 4 (node 18)** | **LHM indirect feedlot for LHM backgrounders using strategy* 1 (node 19)** | **HHM indirect feedlot for LHM backgrounders using strategy* 4 (node 24)** | **HHM indirect feedlot for HHM backgrounders using strategy* 2 (node 25)** |
|  | Per animal basis | | | | | |
| Feeder weight (kg) | 318 | 318 | 415 | 318 | 318 | 415 |
| Days in phase (days) | 177 | 213 | 162 | 177 | 247 | 127 |
| ADG per day (kg) | ***1.60*** | ***1.40*** | ***1.10*** | ***1.60*** | ***1.10*** | ***1.50*** |
| Death loss during phase (%) | ***1.50%*** | ***3.00%*** | ***0.75%*** | ***1.50%*** | ***0.75%*** | ***2.00%*** |
| Final node weight (kg) | 590 | 590 | 590 | 590 | 590 | 590 |
| **Feedlot expenses (per head)** |  |  |  |  |  |  |
| Vet/vaccine/drugs | $17 | $11 | $4 | $17 | $4 | $15 |
| Labor | $29 | $29 | $16 | $29 | $16 | $30 |
| Marketing | $19 | $21 | $18 | $19 | $18 | $20 |
| Fuel and energy | $24 | $27 | $20 | $24 | $20 | $24 |
| Machinery | $22 | $23 | $20 | $22 | $20 | $22 |
| Interest on cattle loan/Other | $37 | $31 | $24 | $37 | $24 | $35 |
| Fixed expense | $49 | $115 | $141 | $49 | $141 | $87 |
| **Feed Expense** |  |  |  |  |  |  |
| Harvested forage cost | $19 | $20 | $79 | $19 | $79 | $22 |
| Grain/supplement | $221 | $236 | $221 | $221 | $221 | $249 |
| Mineral | $20 | $21 | $20 | $20 | $20 | $23 |
| Total expenses during the phase | ***$457*** | ***$534*** | ***$563*** | ***$457*** | ***$563*** | ***$527*** |
| Herd level variables |  |  |  |  |  |  |
| Total cost of production for node | $1,051,843,963 | $1,231,181,822 | $1,296,041,463 | $1,051,843,963 | $1,296,041,463 | $1,213,017,979 |
| Total initial weight (metric ton) | 356,142 | 356,142 | 2,826,301 | 356,142 | 356,142 | 2,826,301 |
| Total final weight (metric ton) | 660,335 | 659,874 | 4,016,927 | 660,335 | 659,336 | 4,020,011 |
| **Node cost^1^ (per ton of weight gain)** | **$3,458** | **$4,054** | **$1,089** | **$3,458** | **$4,275** | **$1,016** |
| **Weight gain coefficient^2^** | **185%** | **185%** | **142%** | **185%** | **185%** | **142%** |

*All the 6 groups had 3 feedlots (so 18 direct feedlots^#^), each of which followed a specific antibiotic use management strategy out of 4 possible strategies, namely 1) Metaphylaxis along with treatment of sick animals (feedlots receiving high quality health feeder cattle couldn't use this strategy), 2) Treatment of sick animals only (feedlots receiving low quality health feeder cattle couldn't use this strategy), 3) Feedlots in which any use of antibiotic use was banned, 4) Feeder cattle are exclusively grass-grazing and antibiotic use was banned

^#^The other 12 out of a total of 18 different (nodes 11-13, 15-17, 20-23, 26, 27) node costs and weight gain coefficients for indirect feedlots used in the optimization model are available in table 1

^1^Node cost per metric ton of weight gain for the node 10 is the ratio of total cost of production for node ($1.05 billion) to weight gain (304,193 metric ton) of 1.17 million animals. Same logic used in case of nodes 11-28.

^2^Weight gain coefficient 185% of node 10 is the ratio of total final weight (660,335) and the total initial weight at node (356,142). Same logic used in case of nodes 11-28.
